# Neurogenesis in Neurodegenerative Diseases: Role of MFG-E8

**DOI:** 10.3389/fnins.2019.00569

**Published:** 2019-06-04

**Authors:** Cletus Cheyuo, Monowar Aziz, Ping Wang

**Affiliations:** ^1^Department of Neurosurgery, West Virginia University, Morgantown, WV, United States; ^2^Center for Immunology and Inflammation, The Feinstein Institute for Medical Research, Manhasset, NY, United States; ^3^Department of Surgery and Molecular Medicine, Donald and Barbara Zucker School of Medicine at Hofstra/Northwell, Manhasset, NY, United States

**Keywords:** neurogenesis, neurodegenerative diseases, MFG-E8, Alzheimer’s disease, Parkinson’s disease, stem cells, integrin, apoptosis

## Abstract

Neurodegenerative diseases are devastating medical conditions with no effective treatments. Restoration of impaired neurogenesis represents a promising therapeutic strategy for neurodegenerative diseases. Milk fat globule-epidermal growth factor-factor VIII (MFG-E8) is a secretory glycoprotein that plays a wide range of cellular functions including phagocytosis of apoptotic cells, anti-inflammation, tissue regeneration, and homeostasis. The beneficial role of MFG-E8 has been shown in cerebral ischemia (stroke), neurodegenerative diseases such as Alzheimer’s disease and Parkinson’s disease, and traumatic brain injury. In stroke, MFG-E8 promotes neural stem cell proliferation and their migration toward the ischemic brain tissues. These novel functions of MFG-E8 are primarily mediated through its receptor α_v_β_3_-integrin. Here, we focus on the pivotal role of MFG-E8 in protecting against neuronal diseases by promoting neurogenesis. We also discuss the mechanisms of MFG-E8-mediated neural stem/progenitor cell (NSPC) proliferation and migration, and the potential of MFG-E8 for neural stem cell niche maintenance via angiogenesis. We propose further investigation of the molecular pathways for MFG-E8 signaling in NSPC and effective strategies for MFG-E8 delivery across the blood–brain barrier, which will help develop MFG-E8 as a future drug candidate for the bedside management of neurodegenerative diseases.

## Introduction

Neurodegenerative diseases are a heterogeneous group of brain disorders of multifactorial etiologies characterized by the loss of existing neurons and alterations in neuronal replacement via neurogenesis (De Pablo-Fernández et al., 2019; Zetterberg and Schott, 2019). Common neurodegenerative diseases with alterations in neurogenesis include Parkinson’s disease, Alzheimer’s disease (AD), Huntington disease, Schizophrenia, and Prion disease (Winner and Winkler, 2015; Horgusluoglu et al., 2017; De Pablo-Fernández et al., 2019; Zetterberg and Schott, 2019). These diseases have different clinical manifestations due to different pathological mechanisms occurring in different parts of the brain. However, the ultimate result of the different disease mechanisms at the cellular level is loss of neurons and other cells in the brain. Therefore, pharmacological modulation of the process of neuronal renewal via neurogenesis and inhibition of neuropathological processes is a promising therapeutic strategy (Cheyuo et al., 2015; Hachem et al., 2017).

Milk fat globule-epidermal growth factor-factor VIII (MFG-E8) is a secreted glycoprotein which has two epidermal growth factor (EGF)-like domains from the mouse or one EGF-like domain from human at the N-terminal site, and two discoidin domains at the carboxy terminal site from both (Aziz et al., 2011). Depending on the species and post-translational modifications the molecular weight of MFG-E8 varies from 46 to 75 kD (Aziz et al., 2011). The EGF-like domain has an RGD (Arg-Gly-Asp) motif which recognizes integrins α_v_β_3_ and α_v_β_5_ mainly expressed on macrophages (Hanayama et al., 2002, 2004; Aziz et al., 2011). The discoidin domain of MFG-E8 recognizes phosphatidylserine (PS) exposed on the cell membranes of apoptotic cells (Hanayama et al., 2002, 2004; Aziz et al., 2011). This unique structure of MFG-E8 enables it to mitigate several pathological processes involved in neurodegenerative diseases.

MFG-E8-mediated phagocytic clearance of apoptotic cells by the macrophages (Hanayama et al., 2002; Fuller and Van Eldik, 2008; Aziz et al., 2011) is one of the mechanisms by which hyperinflammation in brain diseases can be inhibited. MFG-E8-mediated phagocytosis of prion infected apoptotic cells also helps control prion disease in mice (Kranich et al., 2010). MFG-E8 has been shown to bind amyloid-beta peptide (ABP) and facilitate ABP clearance by the glial cells to protect mice against the development of AD (Boddaert et al., 2007), even though in a mouse model of Parkinson’s disease, MFG-E8 deficiency did not impact of the clearance of apoptotic bodies (Kinugawa et al., 2013). Although phagocytic clearance of apoptotic cells/debris is useful in protecting against neurodegenerative diseases, in the current review we will primarily focus on the plausible role of MFG-E8 on neurogenesis in neurodegenerative diseases.

MFG-E8 has been shown to promote cell proliferation and cell migration through its receptors α_v_β_3_-integrin and PS (Bu et al., 2007; Aziz et al., 2011; Wang et al., 2012; Cheyuo et al., 2015). Therefore, besides phagocytic clearance of apoptotic cells or debris, proliferation and migration of stem cells as reported in murine cerebral ischemia (Cheyuo et al., 2015), regeneration of injured tissues, and mitigation of overall inflammation as mediated by MFG-E8 provide strong scientific ground to establish MFG-E8 as a promising candidate therapeutic agent for neurodegenerative diseases.

## Neurogenesis

During early corticogenesis [embryonic day 9 (E9) to E11 in mice], neural stem/progenitor cells (NSPCs) expand clonally through symmetrical cell division, producing two similar self-renewing daughter cells (Haubensak et al., 2004; Radakovits et al., 2009; Tsunekawa and Osumi, 2012). From E11, NSPC transform morphologically into thin, elongated, asymmetrically dividing cells called radial glial cells (RGL; Noctor et al., 2001; Götz and Huttner, 2005). Asymmetrical division produces two daughter cells with different fates – an apical progenitor (AP) with self-renewing capacity through further asymmetrical divisions, and an intermediate progenitor (IP) which divides symmetrically to produce two daughter cells that undergo differentiation into neurons (Noctor et al., 2001; Götz and Huttner, 2005; Tsunekawa and Osumi, 2012). RGL support the migration of neuroblasts to the outer layers of the developing cortex where they differentiate into neurons (Götz and Huttner, 2005). The dynamic clonal lineage progression from NSPC to neurons may involve at least four different cell states, namely – quiescent cells, active proliferating cells, transient amplifying progenitors (TAP) and neuroblasts, which ultimately differentiate into neurons after migration (Bast et al., 2018). Neurogenesis is initiated in the neural stem cell niche from the quiescent cells, which upon activation go into a proliferative phase, followed by migration of neuroblasts and eventually differentiation into neurons (Alvarez-Buylla and Garcia-Verdugo, 2002). In the adult brain, neurogenesis is maintained in the subventricular zone and dentate gyrus, which contain neural stem cell niches (García-Verdugo et al., 1998; Doetsch et al., 1999; Bast et al., 2018). The process of neurogenesis holds therapeutic potential for brain diseases characterized by neuronal cell loss.

## MFG-E8 and Neurodegenerative Diseases

Neurodegenerative diseases are irredeemable and devastating clinical conditions that result in the degeneration and/or death of neuronal cells (Winner and Winkler, 2015; Zetterberg and Schott, 2019). Examples of neurodegenerative diseases with alterations in neurogenesis include Parkinson’s disease, Alzheimer’s disease, Huntington disease, Schizophrenia and Prion disease (Winner and Winkler, 2015; Horgusluoglu et al., 2017; De Pablo-Fernández et al., 2019; Zetterberg and Schott, 2019). Parkinson’s disease is characterized pathologically by intracellular deposits of α-synuclein and degeneration of neurons (Schulz-Schaeffer, 2010). The disease process affects the hippocampus and olfactory bulb early (Kohl et al., 2012). Decreased neurogenesis is observed in both animal models of Parkinson’s disease and human post-mortem studies (Höglinger et al., 2004; Kohl et al., 2012). Alzheimer’s disease is a common type of dementia, characterized pathologically by neurofibrillary tangles and amyloid plaques, with widespread degenerative changes involving the basal forebrain and limbic system (Hardy and Selkoe, 2002; Zetterberg and Schott, 2019). Hippocampal neurogenesis has been shown to be impaired in Alzheimer’s disease (Sun et al., 2009; Mu and Gage, 2011; Zetterberg and Schott, 2019). In Huntington disease, there is a trinucleotide expansion within the Huntington gene due to an autosomal dominant mutation (Pringsheim et al., 2012). The proliferation of neural stem cells is not affected in Huntington disease but there is impaired maturation of neurons in the striatum (Phillips et al., 2005; Winner and Winkler, 2015). Schizophrenia is another common neurodegenerative disorder characterized clinically by delusions, hallucinations, thought disorder, and movement disorder. Cognitive impairment in schizophrenia is correlated with disruption of neurogenesis (Reif et al., 2006; Lee et al., 2015). Prion disease, also known as Creutzfeldt-Jakob disease in humans, is a fatal neurodegenerative disease caused by host-encoded cellular prion protein (PrPC) misfolding into infectious disease-provoking multimeric aggregates, called prions (Kupfer et al., 2009). The prions are capable of infecting neural stem cells and altering neuronal destiny during neurogenesis (Relaño-Ginés et al., 2014). Most of the current treatments of neurodegenerative diseases only relieve symptoms or slow down disease progression, without a true cure. Given the progressive nature of these diseases, with continuing neuronal loss, there comes a time when the treatments become ineffective or side effects become unbearable with dose escalation. To counter the loss of neurons with disease progression, induction of neurogenesis could be a promising therapeutic strategy in neurodegenerative diseases (Cheyuo et al., 2015; Hachem et al., 2017).

MFG-E8 is expressed by a wide variety of cells, including immune cells, astrocytes, microglia and neural stem cells, mesenchymal stem cells and hematopoietic stem cells (Aziz et al., 2011; Cheyuo et al., 2012, 2015). MFG-E8 signaling in these cells has been shown to promote apoptotic cell clearance, suppress inflammation, and induce neurogenesis (Aziz et al., 2011; Cheyuo et al., 2015). The anti-inflammatory and anti-apoptotic roles of MFG-E8 have been described in various animal models of brain injury (Cheyuo et al., 2012, 2015; Gao et al., 2018). In rodent model of cerebral ischemia, it has been shown that MFG-E8 inhibits inflammasome-induced production of interleukin-1β (IL-1β) by macrophages (Deroide et al., 2013). MFG-E8 promotes this anti-inflammatory effect via the interaction of integrin β_3_ and P_2_X_7_ receptors (Deroide et al., 2013). In a separate study, MFG-E8 was also found to significantly reduce the expression of the inflammatory cytokine, IL-6, after cerebral ischemia (Cheyuo et al., 2012). In addition, MFG-E8 also inhibits neuronal apoptosis by increasing increased bcl-2/bax ratio (Cheyuo et al., 2012). Bok et al. (2017) found that the MFG-E8 gene was a target of hypoxia-inducible factor-1α (HIF-1α), which helps regulate microglial functions affecting neuronal survival in ischemic stroke. In an animal model of subarachnoid hemorrhage, MFG-E8 reduced neuronal cell death via decreased expression of cleaved caspase-3 and IL-1β (Liu et al., 2015). These studies highlight the potential to ameliorate acute brain injury. Recent studies have also revealed an emerging role of MFG-E8 in regenerative brain repair process of neurogenesis (Cheyuo et al., 2015; Zhou et al., 2018), which shed lights on the development of MFG-E8 as a novel enhancer of neurogenesis in neurodegenerative diseases. By inhibiting the deleterious processes of neuro-inflammation and apoptosis while enhancing neuronal regeneration as described above, MFG-E8 has the potential to be developed as a therapeutic agent for neurodegenerative diseases.

## Expression and Role of MFG-E8 Receptor, α_v_β_3_-Integrin, in NSPC

Crucial to the development of MFG-E8 as a modulator of neurogenesis is the question of whether or not its receptor, α_v_β_3_ integrin, is expressed in NSPC? Integrins are a family of receptors, consisting of heterodimers of α- and β-subunits, which control cellular processes by binding to extracellular matrix (ECM), soluble extracellular ligands or cell surface molecules (Berrier and Yamada, 2007). Integrin signaling is critical for corticogenesis through the regulation of neurogenesis (Fietz et al., 2010). Mammalian integrins consist of 8 β-subunits and 16 α-subunits, which can combine to generate 24 known functional receptor types (Prowse et al., 2011). In NSPC, the β_1_-subunit is the most commonly expressed integrin subunit, being expressed by 94% of human NSPC (Flanagan et al., 2006). The MFG-E8 receptor, α_v_β_3_ integrin, is expressed in human, ferret and mouse NSPCs (Fietz et al., 2010; Stenzel et al., 2014). RGL, which divide asymmetrically, are elongated NSPC with polarity maintained by differential expression of molecules in their basal and apical processes. Fietz et al. (2010) found that β_3_-integrin was expressed in the basal processes of asymmetrically dividing NSPC and that disruption of its function resulted in decreased expansion. Similarly, Stenzel et al. (2014) demonstrated that activation of α_v_β_3_-integrin promoted NSPC expansion in the mouse embryonic neocortex. The crucial role of α_v_-integrin in mouse brain development was characterized by McCarty et al. (2002) in α_v_-null mice. These mice were found to develop intracerebral hemorrhage mid-gestation and die shortly after birth. These knockout mice displayed normal endothelium-pericyte associations and inter-endothelial cell junctions. However, the cause of hemorrhage was revealed to be defective associations between cerebral micro-vessels and the surrounding brain parenchyma, composed of neuroepithelial cells, glia, and neuronal precursors. In addition, these mice also developed disorganized neuroepithelial processes in their ganglionic eminences. Integrin β_3_ and β_5_ knockout mice on the other hand, did not cause cerebral hemorrhages (McCarty et al., 2002). Of note, β_5_-integrin expression has not been characterized in neural stem cells (Prowse et al., 2011). Using the Cre/Lox system, McCarty et al. (2005) further characterized the effect of conditional deletion of α_v_-integrin in central nervous system glia and neurons. Mice with this conditional deletion of α_v_-integrin also developed cerebral hemorrhage. Surviving mice subsequently developed severe neurological deficits and seizures and died by age 4 weeks (McCarty et al., 2005). On the other hand, integrin β_3_ knockout in mice is not lethal (Carter et al., 2011). Integrin β_3_ knockout mice have been found to display altered social and repetitive behavior relevant to autism spectrum disorders (Carter et al., 2011). In summary, the above studies have demonstrated the pivotal role of the MFG-E8 receptor, α_v_β_3_ integrin, in NSPC expansion and maintenance of the associations between cerebral micro-vessels and the surrounding brain parenchyma.

## α_v_β_3_-Integrin-Mediated Signal Transduction Pathway

The signal transduction pathways through which the α_v_β_3_-integrin receptor mediates its myriad cellular effects are complex and are covered in great detail elsewhere in the literature (Desgrosellier et al., 2009; Schiller and Fässler, 2013; Xiao et al., 2013). Briefly, engagement of the α_v_β_3_-integrin with its ligands causes clustering of the integrin in the membrane and concentration of intracellular signaling molecules (Berrier and Yamada, 2007). Clustering of the β_3_-integrin intracellular domain activates non-receptor tyrosine kinases such as focal adhesion kinase (FAK) and Src, resulting in increased concentrations of tyrosine-phosphorylated proteins (Berrier and Yamada, 2007; Desgrosellier et al., 2009). Different types of proteins with different functions are recruited to the integrin receptor activation complex. These proteins may include scaffolding proteins, such as talin, which link integrins to cytoskeletal components such as actin, α-actinin and vinculin, leading to stimulation of cell migration (Berrier and Yamada, 2007). Serine/threonine kinases, such as phosphoinositide 3 kinase (PI3K), have also been shown to be recruited to integrin receptor activation complex, where they interact with FAK and Src, leading to the downstream regulation of the cell cycle via regulation of cyclins and cyclin dependent kinases (Schwartz and Assoian, 2001). The specific aspects of the complex α_v_β_3_-Integrin-mediated signal transduction pathway activated by MFG-E8 are discussed in the later part of the review.

## Neural Stem Cell Niche

Neurogenesis in the postnatal brain is restricted to the subventricular zone and the dentate gyrus, which contain niches where NSPC reside (Alvarez-Buylla and Garcia-Verdugo, 2002). The neural stem cell niche is a specialized unit consisting of capillaries in planar configuration, in direct contact with NSPC. The unique neurovascular interface of the niche lacks the typical endothelial tight-junctions, astrocyte endfeet and pericytes that make up the typical blood–brain barrier (BBB; Tavazoie et al., 2008). Indeed, tracer experiments demonstrated high tracer uptake at the subventricular zone, confirming a leaky BBB at the neural stem cell niche (Tavazoie et al., 2008). This suggests that small molecules in the circulation may be able to cross this barrier to modulate neurogenesis. The neural stem cell component of the niche consists of NSPC in different stages of the cell cycle (Bast et al., 2018). Quiescent neural stem cells are cells in the G_0_ phase that retain the ability to re-enter the cell cycle and contribute to cell proliferation (Daynac et al., 2013). Proliferating cell populations in the neural stem cell niche include active proliferating neural stem cells, TAP and proliferating neuroblasts (Bast et al., 2018). Non-proliferating neuroblasts are committed progenitors which migrate out of the niche to other parts of the brain where they undergo differentiation (Thored et al., 2007).

NSPC in the niche expand by two main types of cell division – asymmetric and symmetric cell divisions (Bast et al., 2018). Symmetric cell division produces two similar self-renewing daughter cells. Asymmetric cell division on the other hand produces two daughter cells with different fates – one daughter cell is self-renewing while the other daughter cell becomes committed to differentiation (Tsunekawa and Osumi, 2012). From E-11, NSPC transform morphologically into thin, elongated, asymmetrically dividing cells called RGL. Each RGL has a basal process and an apical process (Noctor et al., 2001; Götz and Huttner, 2005). RGL are molecularly polarized, with different patterns of protein expression profiles between the basal and apical processes (Noctor et al., 2001; Götz and Huttner, 2005). Molecules distributed in the basal process include cyclin D2 and integrins, which interact with ECM molecules in the basal lamina. Apically distributed molecules include centrosomal proteins, cell adhesion molecules and prominin 1 (Tsunekawa and Osumi, 2012; Tsunekawa et al., 2014). The basally biased location of cyclin D2, a cell cycle regulator, results in asymmetric inheritance of cyclin D2 after asymmetric cell division, leading to cell fate determination. In a model of cell fate determination described by Tsunekawa et al. (2014), cyclin D2 mRNA is transported to the basal endfoot during G_1_, S- to G_2_-phase due to the *cis*-transport element that resides in the 3′ UTR region of cyclin D2 mRNA together with the transportation machinery that recognizes the *cis* element. The transported mRNA is locally translated into protein via ribosomes localized at the basal endfoot (Tsunekawa et al., 2014). During mitosis, cyclin D2 protein is inherited by one of the daughter cells with its basal process. The daughter cell that has inherited cyclin D2 with the basal process remains as a progenitor, whereas the other daughter without the cyclin D2 proceeds to differentiation (Tsunekawa and Osumi, 2012; Tsunekawa et al., 2014). The behavior of NSPC in the niche are complex and poorly understood. Bast et al. (2018) performed *in vivo* clonal lineage analysis in double hemizygous GLAST^CreERT2^:Confetti transgenic mice and used a mathematical model to quantify lineage transitions in young and aged mice. They found that asymmetric division was the dominant mode of NSPC division with aging and was the main driver of NSPC quiescence (Bast et al., 2018).

## MFG-E8-Mediated Induction of Neurogenesis

MFG-E8 and its receptor, α_v_β_3_-integrin, are expressed by NSPC, thus suggesting a pivotal role of MFG-E8 in the regulation of neurogenesis (Fietz et al., 2010; Stenzel et al., 2014; Cheyuo et al., 2015; Zhou et al., 2018). A recent study demonstrated the effects of MFG-E8 on neurogenesis, using a rodent model of ischemic stroke (Cheyuo et al., 2015). Stroke was induced in wild-type (WT) and MFG-E8-deficient (MFG-E8^−/−^) mice by transient middle cerebral artery occlusion (tMCAO; Cheyuo et al., 2015). They found that treatment of recombinant human MFG-E8 significantly improved the neurological deficit score, body weight loss and neural stem cell proliferation after tMCAO. Conversely, decreased neural stem cell proliferation was observed in MFG-E8^−/−^ mice in comparison with the WT counterparts which underwent tMCAO (Cheyuo et al., 2015). Recombinant murine MFG-E8 stimulated the proliferation of mouse embryonic neural stem cells via upregulation of cyclin D2 and downregulation of p53, through α_v_β_3_-integrin signaling (Cheyuo et al., 2015). Recombinant murine MFG-E8 also promoted mouse embryonic neural stem cell migration via α_v_β_3_-integrin dependent upregulation of netrin-1 (Cheyuo et al., 2015).

**FIGURE 1:**
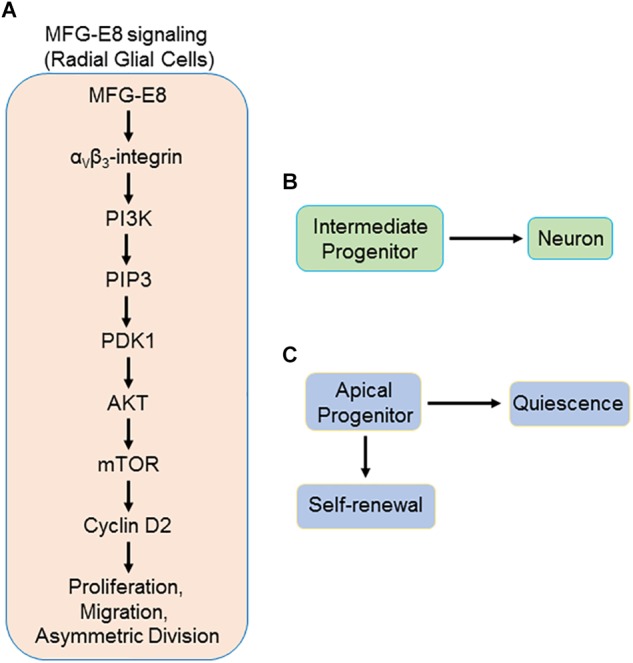
Schematic representation of proposed MFG-E8 signaling in radial glial cells (RGL) via α_v_β_3_/PI3K/Akt/mTOR pathway. **(A)** MFG-E8 binds to α_v_β_3_-integrin on the basal process of RGL, leading to activation of PI3K. PI3K phosphorylates PIP2 and produces PIP3. PIP3 then activates PDK1 which subsequently activates Akt by phosphorylation. Phosphorylation of Akt activates mTOR. mTOR activates several transcription factors leading to the downstream activation of several target genes, including CCDN2, which produces cyclin D2 mRNA. Cyclin D2 mRNA is transported to the basal process by a carrier protein that recognizes its *cis*-transport element that resides in the 3′ UTR region. The mRNA is then translated in the basal process by ribosomes into cyclin D2 protein. Asymmetric division of RGL leads to the production of two daughter cells. **(B)** Intermediate progenitor, with no cyclin D2, which subsequently undergoes differentiation into a neuron, and **(C)** apical progenitor, which has inherited the cyclin D2. This apical progenitor has the capacity for self-renewal through further asymmetric divisions. On the other hand, depending on additional signals received, this RGL may go into quiescence. MFG-E8, milk fact globule-epidermal growth factor-factor VIII; RGL, radial glial cells; PIP2, phosphatidylinositol 3,4-bisphosphate; PIP3, phosphatidylinositol 3,4,5-trisphosphate; PDK1, 3-phosphoinositide-dependent kinase 1; mTOR, mammalian target of rapamycin.

Cyclin D2 is the only D-type cyclin expressed in NSPC, thus making it essential for NSPC biology (Kowalczyk et al., 2004). Tsunekawa et al. have shown that cyclin D2 is a basal cell-fate determinant during asymmetrical cell division in NSPC as described above (Tsunekawa and Osumi, 2012; Tsunekawa et al., 2014). Cyclin D2 knockout mice have been shown to have suppressed neurogenesis and pharmacological suppression of cyclin D2 expression has also been shown to decrease the proliferation of neural stem cells (Garthe et al., 2014; Kerever et al., 2014). MFG-E8 has been shown to positively regulate cyclin D2 expression in neural stem cells (Cheyuo et al., 2015). The upstream pathway involving MFG-E8’s receptor, α_v_β_3_-integrin, might activate unknown downstream molecules which govern cyclin D2 expression in neural stem cells (Cheyuo et al., 2015). MFG-E8 has been shown to activate PI3K/Akt pathway (Zhao et al., 2017; Gao et al., 2018). Moreover, recent studies have also revealed the MFG-E8-mediated upregulation of mammalian target of rapamycin (mTOR; Khalifeh-Soltani et al., 2014; Gao et al., 2018). Cyclin D2 is one of the target genes of mTOR (Balcazar et al., 2009). Therefore, MFG-E8 signaling via α_v_β_3_-integrin could potentially activate the PI3K/Akt/mTOR pathway leading to upregulation of cyclin D2 expression and asymmetrical division in NSPC. The potential induction of asymmetrical division of NSPC by MFG-E8-induced upregulation of cyclin D2, could also drive both proliferation and quiescence of NSPC, deducing from the finding by Bast et al. (2018) that asymmetrical division is the main driver of NSPC quiescence. Beside MFG-E8-mediated α_v_β_3_ integrin signal transduction in NSPC, Zhou et al. (2018) recently described a role for MFG-E8 signaling via α_8_β_1_-integrin signaling. The β_1_-integrin subunit is the predominant integrin expressed in NSPC (Flanagan et al., 2006). The α_8_-integrin subunit and its association with β_1_-subunit was first described in neurons and to some extend in epithelial cells, in chick embryos (Bossy et al., 1991). MFG-E8 signaling through this receptor was reported to promote RGL quiescence through downregulation of mTOR1 (Zhou et al., 2018). A proposed model demonstrating the findings of these recent studies on the novel role of MFG-E8 in neurogenesis has been presented in Figure 1.

## MFG-E8-Mediated Angiogenesis

MFG-E8 is a multi-functional glycoprotein known to mediate other cellular functions other than its typical role in phagocytosis and anti-inflammation (Aziz et al., 2011; Li et al., 2013). MFG-E8 has been shown to promote post-ischemic neovascularization via α_v_β_3_- and α_v_β_5_-dependent Akt phosphorylation and vascular endothelial growth factor (VEGF) induction in endothelial cells (Silvestre et al., 2005). MFG-E8 has also been shown to promote wound healing by stimulating vascular capillary formation (Uchiyama et al., 2014). Uchiyama et al. (2014) found that in the dermis of normal murine and human skin, accumulations of MFG-E8 were found around CD31 expressing blood vessels, and MFG-E8 co-localized with PDGFRβ(+), αSMA(+), and NG2(+) pericytes. They found that MFG-E8 increased capillary formation and myofibroblast recruitment, leading to increased wound healing (Uchiyama et al., 2014). Planar capillaries are an integral part of the neural stem cell niche, as described above (Tavazoie et al., 2008). The endothelial cells secrete trophic factors that support NSPC proliferation and differentiation. In addition, it has also been shown that direct cell to cell interaction between NSPC and endothelial cells promote quiescence through ephrinB2 and Jagged1 signal-transduction pathways (Ottone et al., 2014). Age-related microvascular disease has been shown to contribute to decline in neurogenesis and dementia (Apple and Kokovay, 2017). Thus, in addition to modulating NSPC activity, MFG-E8 may also contribute to maintaining the neural stem cell niche by promoting angiogenesis.

## MFG-E8 Delivery Into the Brain

Even though the BBB at the neural stem cell niche is leaky, as described above (Tavazoie et al., 2008), it is not clear whether intravenously administered MFG-E8 can reach the neural stem cell niche. Indeed, Falborg et al. (2010) assessed the biodistribution of intravenously administered radio-labeled MFG-E8 (99m Tc-HYNIC-lactadherin) and found that there was no uptake in the brain. In a recent study of the effect of MFG-E8 on neurogenesis, the BBB was bypassed by delivering MFG-E8 to the brain via intracerebroventricular administration (Cheyuo et al., 2015). Intracerebroventricular administration of drugs is an invasive route of drug delivery which would not be suitable for long-term treatment of chronic neurodegenerative diseases. Neurogenesis involves the proliferation of NSPC in the neural stem cell niche, where the BBB is leaky, followed by the migration of NSPC out of the niche to distant parts of the brain, with intact BBB, for differentiation. Thus, the development of an effective therapeutic strategy for stimulating neurogenesis in neurodegenerative diseases will require the non-invasive or minimally invasive delivery of an agent that can reach both the neural stem cell niche via the leaky BBB and also be able to cross the intact BBB to influence migration and differentiation of NSPC. The pharmacokinetics of intravenously administered lactadherin (MFG-E8) was studied by Poulsen et al. (2013) in pigs. Even though the effective half-life of MFG-E8 was not determined, they found that a large percentage of intravenously administered MFG-E8 was rapidly sequestered in the liver (Poulsen et al., 2013). Thus, intravenous administration of MFG-E8 for the long-term treatment of chronic neurodegenerative disease would require daily multiple injections, which would be associated with significant patient discomfort and therefore increased non-compliance.

Exosomes are 30–100 nm vesicles formed within endosomes in cells and released into the extracellular space (Conde-Vancells et al., 2008; Colombo et al., 2014; Vogel et al., 2018). Exosomes contain a variety of molecules such as microRNAs, cell membrane proteins and adhesion molecules, which allow them to modulate cellular activity (Conde-Vancells et al., 2008). The membranes of exosomes contain lipids such as phosphatidylserine, sphingomyelin, and phosphatidylcholine (Subra et al., 2007). Exosomes have the advantage of being able to cross the BBB (Qu et al., 2018; Tian et al., 2018). Other advantages of exosomes as a therapeutic delivery tools include the fact that isolated exosomes can be engineered by loading them with the desired therapeutic agent (Qu et al., 2018). Moreover, exosomes can be delivered non-invasively via the intranasal route (Long et al., 2017). The binding of MFG-E8 to phosphatidylserine allows MFG-E8 to be isolated with exosomes. MFG-E8-enriched exosomes from dendritic cells have previously been isolated and used for the treatment of experimental sepsis (Miksa et al., 2009). Because of the universal presence of phosphatidylserine in all cell membranes, even exosomes isolated from cells which do not express MFG-E8 can also be engineered by loading them with MFG-E8 through its binding to the phosphatidylserine. Exosomal MFG-E8 delivery across the BBB has not been studied. However, given the fact that exosomes can effectively cross the BBB, we would expect that MFG-E8 can be delivered to the brain via exosomal transport. Thus, we propose that exosomal MFG-E8, administered non-invasively via the intranasal route or intravenously, could be a long-term delivery strategy for stimulating neurogenesis for therapeutic purposes in neurodegenerative diseases.

**FIGURE 2:**
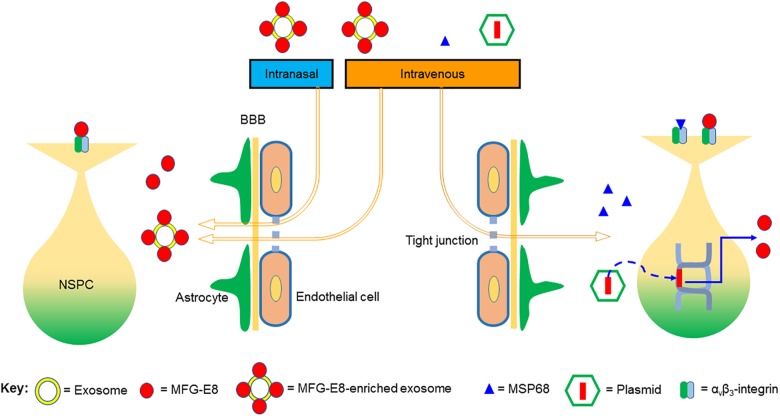
Proposed strategies for effective delivery of MFG-E8 to neural stem/progenitor cells (NSPCs) in the brain. The blood–brain barrier (BBB) is made up of endothelial tight junctions and astrocyte endfeet. We propose that MFG-E8-enriched exosomes, administered either intranasally or intravenously, can effectively cross the BBB and deliver MFG-E8 to NSPC in the brain. MSP68 is a small peptide derivative of MFG-E8, which we presume would be able to cross the BBB due to its small size. Lastly, MFG-E8 can also be over-expressed in NSPC by plasmid-mediated delivery of the MFG-E8 gene into NSPC. Both MFG-E8 and MSP68 signal through the α_v_β_3_ integrin receptor.

Other therapeutic strategies may include, use of small peptide derivative of MFG-E8, MSP68 (VRGDV), which may have a higher probability of crossing the BBB due to its small size (Yang et al., 2015; Hirano et al., 2017). However, the effect of MSP68 on neural stem cells remains to be investigated. Additional strategies for harnessing the therapeutic potential of MFG-E8 for chronic neurodegenerative may include the development of gene therapy whereby the MFG-E8 gene expression is increased in the neural stem cell niche using recombinant viral technology. The potential strategies for effective delivery of MFG-E8 to NSPC in the brain are summarized in Figure 2.

## Future Perspectives and Conclusion

MFG-E8, which is an endogenous glycoprotein with multifunctional cellular effects, has a great potential for development into a therapy for neurodegenerative diseases. Modulation of neurogenesis is one of the recently discovered functions of MFG-E8. MFG-E8 has been found to promote NSPC proliferation, quiescence, and migration (Cheyuo et al., 2015; Zhou et al., 2018). The effect of MFG-E8 on NSPC differentiation has not been studied. More importantly, the molecular pathways by which MFG-E8 modulates NSPC activity remain to be defined. In addition, further research is needed in establishing strategies for effective delivery of MFG-E8 across the BBB for therapeutic purposes. In this regard, the study of the pharmacokinetics and pharmacodynamics of exosomal preparations of MFG-E8 holds great promise. Currently, only *in vitro* and small animal *in vivo* studies of MFG-E8 effect on neurogenesis have been performed. To accelerate the therapeutic development of MFG-E8 for neurodegenerative diseases, we propose the investigation of the effects of MFG-E8 on neurogenesis in larger gyrencephalic animals, whose embryonic corticogenesis and adult neuro-architecture are similar to humans (Sun and Hevner, 2014). Other avenues for harnessing the therapeutic effects of MFG-E8 could be pharmacological upregulation, using agents such as prolactin (Aziz et al., 2008, 2011). In conclusion, the tremendous therapeutic potential of MFG-E8 for neurodegenerative diseases requires further preclinical development.

## Author Contributions

CC, MA did literature review and wrote the manuscript. CC prepared the images. PW reviewed and edited the manuscript. PW conceived the idea of the project.

## Conflict of Interest Statement

One of the authors PW is an inventor of the pending PCT application #WO/2009/064448: “Prevention and treatment of inflammation and organ injury after ischemia/reperfusion using MFG-E8.” This patent application covers the fundamental concept of using MFG-E8 for the treatment of ischemia/reperfusion injury. PW is a co-founder of TheraSource LLC which develops MFG-E8 technology. The remaining authors declare that the research was conducted in the absence of any commercial or financial relationships that could be construed as a potential conflict of interest.
